# Variability in forgoing life-sustaining treatment practices in critically Ill patients with hospital-acquired bloodstream infections: a secondary analysis of the EUROBACT-2 international cohort

**DOI:** 10.1186/s13054-024-05072-1

**Published:** 2024-08-31

**Authors:** Hannah Wozniak, Alexis Tabah, Jan J. De Waele, Jean-François Timsit, Niccolò Buetti, EUROBACT-2 Study Group, the European Society of Intensive Care Medicine (ESICM), the European Society of Clinical Microbiology, the Infectious Diseases (ESCMID) Study Group for Infections in Critically Ill Patients (ESGCIP), the OUTCOMEREA Network

**Affiliations:** 1 Intensive Care Unit, Geneva University Hospitals, Geneva, Switzerland; 2 Faculty of Medicine, Geneva University, Geneva, Switzerland; 3 Intensive Care Unit, Redcliffe Hospital, Brisbane, Australia; 4 Queensland Critical Care Research Network (QCCRN), Brisbane, QLD Australia; 5 Queensland University of Technology, Brisbane, QLD Australia; 6 Faculty of Medicine, The University of Queensland, Brisbane, QLD Australia; 7 Department of Intensive Care Medicine, Ghent University Hospital, Ghent, Belgium; 8 UMR 1137, Université Paris‐ Cité, INSERM, IAME, 75018 Paris, France; 9 Medical and Infectious Diseases Intensive Care Unit, AP‐HP, Bichat‐Claude Bernard University Hospital, Paris, France; 10 Infection Control Program and World Health Organization Collaborating Centre, University Hospitals and Faculty of Medicine, University of Geneva, Geneva, Switzerland

## Abstract

**Background:**

The decision to forgo life-sustaining treatment in intensive care units (ICUs) is influenced by ethical, cultural, and medical factors. This study focuses on a population of patients with hospital-acquired bloodstream infections (HABSI) to investigate the association between patient, pathogen, center and country-level factors and these decisions.

**Methods:**

We analyzed data from the EUROBACT-2 study (June 2019–January 2021) from 265 centers worldwide, focusing on non-COVID-19 patients who died in the hospital or within 28 days after HABSI. We assessed whether death was preceded by a decision to forgo life-sustaining treatment, examining country, center, patient, and pathogen variables. To assess the association of each potentially important variable with the decision to forgo life-sustaining treatment, univariable mixed logistic regression models with a random center effect were performed.

**Results:**

Among 1589 non-COVID-19 patients, 519 (32.7%) died, with 191 (36.8%) following a decision to forgo life-sustaining treatment. Significant geographical differences were observed, with no reported decisions to forgo life-sustaining treatment in African countries and fewer in the Middle East compared to Western Europe, Australia, and Asia. Once a center effect was considered, only health expenditure (Odds ratio 1.79, 95%CI: 1.45–2.21,* p* < 0.01) and age (Odds ratio 1.02, 95%CI: 1.002–1.05, *p* = 0.03) were significantly associated with decisions to forgo life-sustaining treatment, while other patient and pathogen factors were not.

**Conclusion:**

Economic and regional disparities significantly impact end-of-life decision-making in ICUs. Global policies should consider these disparities to ensure equitable end-of-life care practices.

**Supplementary Information:**

The online version contains supplementary material available at 10.1186/s13054-024-05072-1.

## Introduction

Up to 23% of patients die in the intensive care unit (ICU) worldwide, with decisions to forgo (withhold or withdraw) life-sustaining treatment (DFLST) preceding the majority of these deaths [1, 2]. The decision to forgo life-sustaining treatment in ICUs is influenced by a complex interplay of ethical, cultural, and medical factors [2–4]. The Ethicus-2 study, a worldwide study conducted between 2015 and 2016, including 12,850 patients who died or had limitation of treatments, highlighted significant regional variabilities in decisions to forgo life-sustaining treatment. In face of the considerable worldwide variation in end-of-life practices, the authors concluded that future research should investigate the causes of this variation with the aim to improve ethical practices. This was also highlighted in studies conducted in other former worldwide cohorts of various ICU patients [2–7]. Interestingly, a study highlighted that one of the factors associated with more DFLST was septic shock and pneumonia, suggesting that these practices seem to vary according to specific clinical conditions beyond country-level factors [3].

While these studies provide valuable insights, they also reveal the complexity and variability of DFLST due to the heterogeneous nature of the patient populations. By using the EUROBACT-2 cohort consisting of patients all admitted for hospital-acquired bloodstream infections (HABSI), we hypothesized that this approach would allow for a clearer understanding of the specific factors, including country, center, patient, and infection characteristics, that are associated with these critical DFLST.

## Methods

### EUROBACT-2 study design

We conducted a secondary analysis of the data from the EUROBACT-2 study, a prospective observational multicontinental cohort study conducted between June 2019 and January 2021. During this period, centers prospectively recruited patients, with a minimum of 10 consecutive HABSI patients or for a 3-month period [8]. Details on the study process, data collection, and variables can be found elsewhere [8]. Adult patients (≥ 18 years old) with a first episode of HABSI treated in the ICU were enrolled. A HABSI was defined as a positive blood culture sampled 48 h after hospital admission. Treatment in the ICU was defined as either the blood culture having been sampled in the ICU or the patient having been transferred to the ICU (i.e. in 48 h) for the treatment of the HABSI. For this analysis, we focused exclusively on non-COVID-19 patients who died in the hospital or within 28 days after HABSI. We focused on non-COVID-19 patients to avoid the confounding effects of the pandemic, in whom it has been shown that withholding therapies frequently preceded death [9].

The primary outcome of the present study was the occurrence of DFLST preceding death, which was a variable collected prospectively for every death in the EUROBACT-2 cohort.

### Statistical analysis

First, a descriptive analysis of centers, patients' and pathogen’s characteristics according to the decision to forgo life-sustaining treatments was performed. Continuous variables were presented as median with interquartile range (IQR) and categorical variables as number of patients (n) and percentage (%). Chi-square or Fisher's exact tests were used to detect differences in categorical variables, as appropriate, and the Wilcoxon rank-sum test was used for continuous variables.

To identify factors associated with DFLST, we built a two-tiered hierarchical logistic mixed model. The effects of centre-based variables were included as random intercepts. We considered the hierarchical structure of the data, which may manifest as intraclass correlations. We performed univariable mixed logistic regression models to assess the association of each variable with DFLST.

### Ethics

This study was approved by the ethics Committee from the Royal Brisbane & Women's Hospital Human Research (LNR/2019/QRBW/48376). Each study site then obtained ethical and governance approvals according to national and/or local regulations.

## Results

Among the 333 centers recruited in the EUROBACT-2 study, 68 centers were excluded as they either did not include non-COVID-19 patients or did not report any death at day 28. Of the 1589 patients, 519 (32.7%) died, of which 191 (36.8%) had a decision to forgo life-sustaining treatment (Supplementary Table 1, Supplementary Fig. 1).

**Fig. 1:**
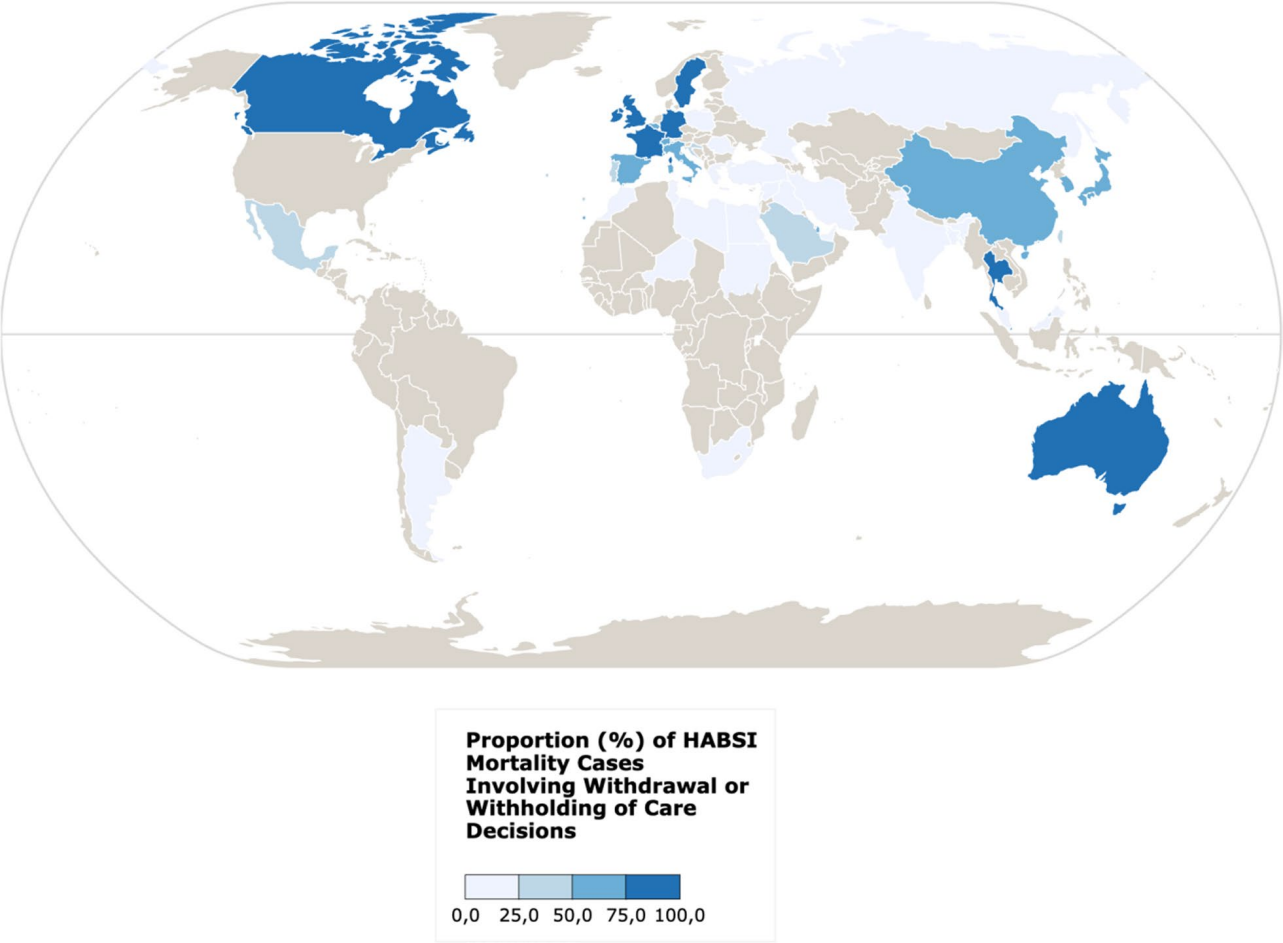
Proportion of hospital-acquired bloodstream infection mortality cases following a decision to forgo life-sustaining treatment. This map highlights the countries that reported Intensive Care Unit (ICU) deaths associated with Hospital-Acquired Bloodstream Infections (HABSI). The map displays the percentage of these deaths that occurred following a clinical decision to forgo life-sustaining treatment. Map created with Khαrtis (https://www.sciencespo.fr/cartographie/khartis/)

The median age was 65 (IQR 54–75), with 204 (39.3%) females. At the country level, significant geographical differences were noted (Fig. 1, Supplementary Table 1), with no DFLST reported in African countries and fewer in the Middle East compared to Western Europe, Australia and Asia. Patients with DFLST were more likely to be from countries with higher current health expenditure as a share of Gross Domestic Product (Supplementary Table 1). At the center level, patients with DFLST were less frequent in centers with open ICUs and higher patient-per-nurse ratios. At the patient level, patients with DFLST were older (median 67, IQR 58–76, vs. 64, IQR 51–75), but other factors such as Charlson comorbidity index, ICU admission reason, infection type, and mechanical ventilation use were not significantly different. No differences in pathogen groups were found between patients with DFLST and those without.

Using univariable mixed logistic regression analysis (Table 1), only gross current health expenditure as a share of Gross Domestic Product (OR 1.79, 95% CI: 1.45–2.21, *p* < 0.01) and older age (OR 1.02, 95% CI: 1.002–1.05, *p* = 0.03) were associated with DFLST.

**Table 1:** Univariate analysis of center, patient, and pathogen factors associated with the decision to forgo life-sustaining treatment

N = 519	DFLST precedes death, OR (95%CI)	*p*-value
*Country/Center factors*		
Current health expenditure as a share of gross domestic product (%)	1.79 (1.45–2.21)	< 0.01
Teaching center	1.19 (0.28–5.09)	0.81
Open ICU^a^	0.31 (0.09–1.06)	0.06
ICU type^a^		
Medical	Ref	
Mixed	0.25 (0.05–1.14)	0.07
Surgical	0.27 (0.03–2.56)	0.25
ICU^a^ funding		
Public	Ref	
Mixed	1.52 (0.09–24.22)	0.76
Private	0.74 (0.13–4.13)	0.74
Patients per nurse ratio	0.88 (0.75–1.03)	0.13
*Patient factor*		
Age, per year	1.02 (1.002–1.05)	0.03
Sex, female	0.86 (0.45–1.63)	0.64
Charlson comorbidity index, per point	1.04 (0.93–1.17)	0.5
Admission type		
Medical	Ref	
Surgical elective	0.84 (0.24–2.94)	0.78
Surgical emergency	1.13 (0.45–2.88)	0.79
Diagnosis of admission		
Cardiovascular disease	Ref	
Respiratory disease	0.96 (0.27–3.40)	0.95
Other	0.98 (0.27–3.61)	0.98
Neurological disease	0.90 (0.27–2.98)	0.86
Abdominal disease	0.22 (0.02–2.97)	0.26
Post-operative care	0.67 (0.17–2.57)	0.46
Septic shock	1.23 (0.05–28.76)	0.89
Source of the HABSI^b^		
Primary	Ref	
Catheter-related	1.02 (0.26–3.93)	0.97
Respiratory	0.46 (0.13–1.62)	0.22
Abdominal	0.59 (0.17–2.09)	0.41
Urinary	0.44 (0.10–1.92)	0.28
Skin	0.58 (0.13–2.57)	0.48
Other	0.24 (0.05–1.26)	0.09
SOFA^c^ on ICU admission, per point	0.96 (0.89–1.05)	0.41
SAPSII^d^ on ICU admission, per point	1.006 (0.98–1.02)	0.46
GCS^e^ on admission, per point	0.95 (0.88–1.02)	0.14
Mechanical ventilation on ICU admission	1.04 (0.79–1.37)	0.76
Dialysis during ICU	0.82 (0.34–2.00)	0.68
Source control:		
-Not required	Ref	
-Required and completed	0.91 (0.45–1.88)	0.82
-Required and not completed	0.57 (0.21–1.57)	0.28
*Infectious factors*		
Gram-positive	0.94 (0.48–1.84)	0.85
Gram-negative	1.03 (0.54–1.96)	0.9
Fungus	0.57 (0.21–1.53)	0.27
Polymicrobial	0.65 (0.22–1.89)	0.43

Results of a univariate mixed-effects logistic regression analysis evaluating the association of center, patient, and pathogen factors with the decision to forgo life-sustaining treatment (DFLST), accounting for center variability

^a^ICU, Intensive care unit

^b^HABSI, Bloodstream infection

^c^SOFA, Sequential organ failure assessment

^d^SAPSII, Simplified acute physiology score II

^e^GCS: Glasgow Coma Scale

## Discussion

These findings, in a worldwide population of critically ill ICU patients with HABSI, highlight the importance of economic and regional disparities on patient outcomes and ethical decision-making in ICUs. Once a center effect was considered, only age and health expenditure were significantly associated with DFLST, underscoring the importance of geographical differences. Interestingly, pathogen factors and source of infection were not associated with DFLST in HABSI patients.

The Ethicus-2 study [2], conducted between 2015 and 2016, highlighted significant regional variability in decisions to forgo life-sustaining treatment. This was also evident in studies conducted in other cohorts, where heterogeneous patient populations, while allowing for generalizing their findings to overall ICU patients, could present more challenges identifying specific factors associated with DFLST [2–7]. Conducted five years after Ethicus-2, our study benefits from including more centers and offers a better representation of African countries. Despite the smaller sample size, it provides a detailed assessment of the variables that matter in terms of DFLST.

Past studies have found that patients' severity upon ICU admission and comorbidities were associated with more DFLST, a finding not observed in our study[3]. Interestingly, our study also found that infection and pathogen factors are not significantly associated with DFLST, reinforcing that variability in these decisions is primarily attributed to center and country effects. This can be explained by several hypotheses, including cultural, religious, and hospital policy differences, emphasizing the need for context-specific guidelines [1–3].

Regarding the importance of age after accounting for a center effect, our results align with previous studies [2, 3]. It appears that even in older patient populations, age continues to matter as a previous study focused on DFLST in old critically ill patients in European countries found that in this specific population, age, frailty, admission SOFA score, and country were the most significant variables associated with decisions to forgo life-sustaining treatments [10].

Our study has several limitations. Due to the sample size, we could not assess for the presence of variabilities in DFLST within a single country, which has been previously highlighted as being important [3]. Additionally, while our data spans multiple countries and centers, due to the limited sample size and secondary analysis nature of the study, it may not fully represent each region's diversity, indicating that further studies with broader representation of centers and populations are warranted. We could not distinguish between withdrawal and withholding of life-sustaining treatment, which might have provided more nuanced insights. The initial code status of the patient upon ICU admission was not available, which could be a significant factor in DFLST when, for example, limited time contracts with patients are made. Lastly, while our study has the advantage of reducing the variability related to different diseases, the heterogeneity of other studies allows for findings to be more generalizable across a broader range of ICU patients and conditions.

In summary, our study underscores the critical impact of regional and economic disparities on ethical decision-making in ICUs worldwide. Future research and global policy development should consider these disparities to enhance equity in end-of-life care practices across different settings.

### Supplementary Information

Additional file1 (DOCX 141 KB)

## Data Availability

The datasets used and/or analysed during the current study are available from the corresponding author on reasonable request.

## Abbreviations

DFLST: Decisions to forgo life-sustaining treatment
ICU: Intensive care unit
HABSI: Hospital-acquired bloodstream infection
