# Supplementary material for: Variability in forgoing life-sustaining treatment practices in critically Ill patients with hospital-acquired bloodstream infections: a secondary analysis of the EUROBACT-2 international cohort
Source: Crit Care. 2024 Aug 31;28:287. doi: 10.1186/s13054-024-05072-1 (PMC11365124; doi:10.1186/s13054-024-05072-1)
Supplement: Supplementary file 1 — Additional file1 (DOCX 141 KB) [file 13054_2024_5072_MOESM1_ESM.docx]

**Supplementary Materials:** Variability in Forgoing Life-Sustaining Treatment Practices in Critically Ill Patients with Hospital-acquired Bloodstream Infections: a Secondary Analysis of the EUROBACT-2 International Cohort

Hannah Wozniak^1,2^, Alexis Tabah^3,4,5,6^, Jan J. De Waele^7^, Jean-François Timsit^8,9^, Niccolò Buetti^2,8,10^, EUROBACT-2 Study Group, the European Society of Intensive Care Medicine (ESICM), the European Society of Clinical Microbiology, the Infectious Diseases (ESCMID) Study Group for Infections in Critically Ill Patients (ESGCIP), and the OUTCOMEREA Network

^1^ Intensive Care Unit, Geneva University Hospitals, Geneva, Switzerland

^2^ Geneva University, Faculty of Medicine, Geneva, Switzerland

^3^ Intensive Care Unit, Redcliffe Hospital, Brisbane, Australia.

^4^ Queensland Critical Care Research Network (QCCRN), Brisbane, QLD, Australia

^5^ Queensland University of Technology, Brisbane, QLD, Australia.

^6^ Faculty of Medicine, The University of Queensland, Brisbane, QLD, Australia.

^7^ Department of Intensive Care Medicine, Ghent University Hospital, Ghent, Belgium.

^8^ Université Paris‐ Cité, INSERM, IAME UMR 1137, 75018 Paris, France.

^9^ Medical and Infectious Diseases Intensive Care Unit, AP‐HP, Bichat‐Claude Bernard University Hospital

^10^ Infection Control Program and World Health Organization Collaborating Centre on Patient Safety, University Hospitals and Faculty of Medicine, University of Geneva, Geneva, Switzerland.

**Supplementary Table 1. Characteristics of Patients Who Died in the Hospital with Hospital-acquired bloodstream infection: Center, Patient, and Pathogen Factors Stratified by Decisions to Forgo Life-Sustaining Treatment**

| N=519 | Death without decision to forgo life-sustaining treatment  N=328 | Death following a decision to forgo life-sustaining treatment  N=191 | p-value |
| --- | --- | --- | --- |
| Country/Center factors | | | |
| Region, n(%)  Americas^1^  Asia^2^  Europe^3^  Middle East^4^  Africa^5^  Oceania^6^ | 8 (2.4%)  59 (18%)  138 (42.1%)  68 (20.7%)  51 (15.5%)  4 (1.2%) | 9 (4.7%)  51 (26.7%)  113 (59.1%)  5 (2.6%)  0 (0%)  13 (6.8%) | <0.01 |
| Current Health Expenditure as a Share of Gross Domestic Product (%), median (IQR) | 6.1 (5.3-7.8) | 9.9 (6.7-11.1) | <0.01 |
| Income of the country^7^, n(%)  High income  Low income  Lower-middle income  Upper-middle income | 139 (42.4%)  3 (0.9%)  54 (16.5%)  132 (40.2%) | 165 (86.4%)  0  1 (0.5%)  25 (13.1%) | <0.01 |
| Academic status of the Hospital, n(%)  Teaching Hospital  Non-teaching Hospital | 283 (86.3%)  45 (13.7%) | 161 (84.3%)  30 (15.7%) | 0.6 |
| ICU^8^ funding, n(%)  Mixed  Private  public | 8 (2.4%)  47 (14.3%)  273 (83.2%) | 7 (3.4%)  23 (12%)  161 (84.3%) | 0.6 |
| ICU^8^ type, n(%)  Medical  Mixed  Surgical | 40 (12.2%)  260 (79.3%)  28 (8.5%) | 40 (20.9%)  130 (68.1%)  21 (11%) | 0.01 |
| Structure of the ICU^8^  Open ICU, n(%)  Closed ICU, n (%) | 101 (30.8%)  227 (69.2%) | 40 (21.1%)  150 (78.9%) | 0.02 |
| Patients per nurse ratio, median (IQR) | 2.3 (1.8-3) | 2 (1.1-2.9) | <0.01 |
| Patient factors | | | |
| Age, median (IQR) | 64 (51-75) | 67 (58-76) | 0.047 |
| Sex, female, n(%) | 132 (40.2%) | 72 (37.7%) | 0.6 |
| Charlson Comorbidity Index, median (IQR) | 2 (1-4) | 2 (1-5) | 0.9 |
| Type of admission, n(%)  Medical  Surgical elective  Surgical emergency | 241 (73.5%)  26 (7.9%)  61 (18.6%) | 144 (75.4%)  13 (6.8%)  34 (17.8%) | 0.9 |
| Diagnosis of admission, n(%)  Cardiovascular disease  Respiratory disease  Other  Neurological disease  Abdominal disease  Post-operative care  Septic shock | 32 (9.8%)  70 (21.3%)  58 (17.7%)  91 (27.7%)  13 (4%)  59 (18%)  5 (1.5%) | 16 (8.4%)  43 (22.5%)  32 (16.8%)  70 (36.7%)  2 (1.1%)  25 (13.1%)  3 (1.6%) | 0.18 |
| Source of the BSI^9^, n(%)  Primary  Catheter-related  Respiratory  Abdominal  Urinary  Skin  Other | 30 (9.2%)  46 (14%)  85 (25.9%)  64 (19.5%)  42 (12.8%)  28 (8.5%)  33 (10.1%) | 21 (11%)  28 (14.7%)  37 (19.4%)  58 (30.4%)  19 (10%)  16 (8.4%)  12 (6.3%) | 0.07 |
| SOFA^10^ on admission, median (IQR) | 11 (8-14) | 10 (7-13) | 0.7 |
| SAPSII^11^ on admission, median (IQR) | 49 (38-63) | 54 (44-69) | <0.01 |
| GCS^12^ on admission, median (IQR) | 13 (8-15) | 13 (6-15) | 0.16 |
| Mechanical ventilation on ICU^8^ admission, n(%) | 213 (65.1%) | 127 (66.5%) | 0.8 |
| Dialysis during ICU^8^, n(%) | 48 (14.6%) | 38 (19.9%) | 0.1 |
| Source control, n(%)  -Not required  -Required and completed  -Required and not completed | 157 (47.9%)  115 (25.1%)  56 (17.1%) | 91 (47.6%)  71 (37.2%)  29 (15.2%) | 0.8 |
| Antibiotic was stopped as part of the for withdrawal of care, n(%) | 24 (11.1%) | 79 (48.5%) | <0.01 |
| Infectious factors | | | |
| Gram-positive, n(%) | 98 (29.9%) | 61 (31.9%) | 0.6 |
| Gram-negative, n(%) | 203 (61.9%) | 113 (59.2%) | 0.6 |
| Fungus, n(%) | 42 (12.8%) | 23 (12%) | 0.9 |
| Polymicrobial, n(%) | 32 (9.76%) | 19 (9.9%) | 0.9 |

^1^ Americas Region: Canada, Mexico, Argentina; ^2^ Asia Region: China, India, Japan, South Korea, Malaysia, Singapore, Thailand, Taiwan, Bangladesh; ^3^ Europe Region: Belgium, Germany, Spain, France, United Kingdom, Greece, Croatia, Italy, Poland, Portugal, Romania, Russia, Switzerland, Ireland, Sweden; ^4^ Middle East Region: Iran, Qatar, United Arab Emirates, Iraq, Israel, Lebanon, Saudi Arabia, Syria; ^5^ Africa Region: Egypt, Libya, Morocco, Tunisia, Niger, Sudan, South Africa; ^6^ Oceania Region: Australia; ^7^ based on the World Bank Income Classifications; ^8^ICU, Intensive Care Unit; ^9^BSI, Bloodstream Infection;^10^SOFA, Sequential Organ Failure Assessment; ^11^SAPSII, Simplified Acute Physiology Score II; ^12^GCS: Glasgow Coma Scale

**Supplementary Figure 1. Consort Diagram**


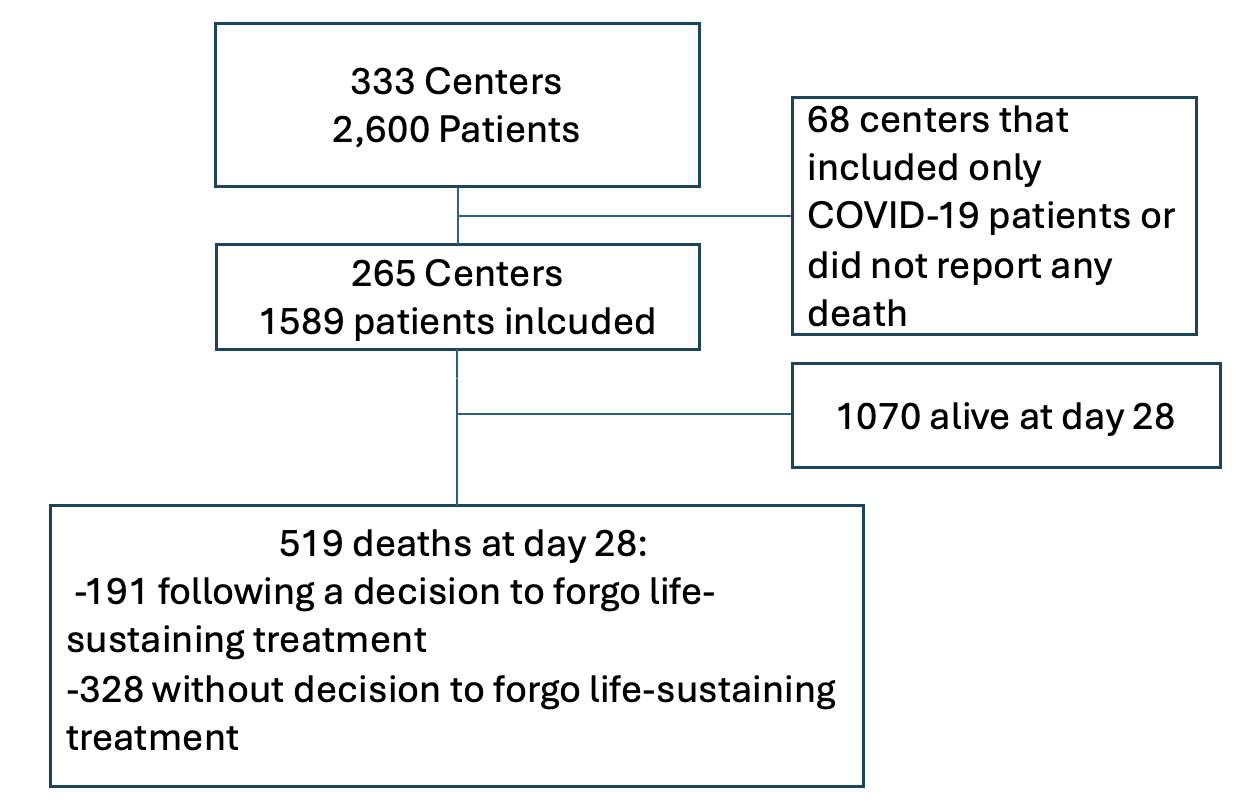


**The Eurobact-2 study group: National coordinators, scientific committee, and participating intensive care units.**

**East Asia and Pacific**

**Australia**

*National Coordinator:* A/Prof. Alexis Tabah

*Scientific Committee:* Prof. Jeffrey Lipman

*Participating ICUs:* Redcliffe Hospital, ICU: A/Prof. Alexis Tabah, Dr Hamish Pollock, Dr Ben Margetts. Alfred Hospital, Department of Intensive Care and Hyperbaric Medicine: Prof Andrew Udy, Ms Meredith Young. Ipswich Hospital, Intensive Care Unit: Dr Neeraj Bhadange, Mr Steven Tyler. Mater Hospital, And Mater Research Institute – The University of Queensland, Mater Misericordiae Limited, The Department of Intensive Care: Dr Anne Ledtischke, Miss Mackenzie Finnis. Mater Private Hospital, And Mater Research Institute – The University of Queensland, Mater Misericordiae, The Department of Intensive Care: Dr Anne Ledtischke, Miss Mackenzie Finnis. Bankstown-Lidcombe Hospital, Intensive Care Unit: Dr Jyotsna Dwivedi, Dr Manoj Saxena. Lyell Mcewin Hospital, Lyell Mcewin Hospital Intensive Care Unit: Dr Vishwanath Biradar, Mrs Natalie Soar. Cabrini Hospital, Intensive Care: A/Prof Vineet Sarode, A/Prof David Brewster. St John Of God Murdoch Hospital, Intensive Care Unit: A/Prof Adrian Regli, Dr Elizabeth Weeda. Royal Brisbane and Women S Hospital, Intensive Care Services: Dr Samiul Ahmed, Ms Cheryl Fourie, Prof. Kevin Laupland. The Prince Charles Hospital, Adult Intensive Care Services: Dr Mahesh Ramanan. Princess Alexandra Hospital, Intensive Care: Dr James Walsham, Mr Jason Meyer. Fiona Stanley Hospital, Intensive Care Unit: Dr Edward Litton, Ms Anna Maria Palermo, Mr Timothy Yap, Mr Ege Eroglu. Rockhampton Hospital, Intensive Care Unit: Dr Antony George Attokaran, Dr C'havala Jaramillo.

**Brunei**

*National Coordinator:* Dr. Khalid Mk Nafees

*Participating ICUs:* Ripas Hospital, Icu 3: Dr Khalid Mahmood Khan Nafees. Raja Isteri Pengiran Anak Saleha Hospital, Icu1: Dr Nurhikmahtul Aqilah Haji Abd Rashid, Dr Haji Adi Muhamad Ibnu Walid. Gleneagles Jpmc, Icu: Dr Tomas Mon, Dr P. Dhakshina Moorthi. Suri Seri Begawan Hospital, Intensive Care Unit: Dr Shah Sudhirchandra, Dr Dhadappa Damodar Sridharan.

**China**

*National Coordinator:* Dr. Qiu Haibo and Dr. Jianfeng Xie

*Participating ICUs:* Zhongda Hospital, Southeast University, Department of Critical Care Medicine: Dr Qiu Haibo, Dr Xie Jianfeng. Yijishan Hospital, First Affiliated Hospital of Wannan Medical College, Department of Critical Care Medicine: Dr Lu Wei-Hua, Dr Wang Zhen. First Affiliate Hospital of Kunming Medical University, Micu/Eicu: Prof Chuanyun Qian, Dr Jili Luo. Qilu Hospital of Shandong University, Department of Critical Care Medicine: Dr Xiaomei Chen, Dr Hao Wang. Hebei Petrochina Central Hospital, Intensive Care Unit: Dr Peng Zhao, Dr Juan Zhao. Hangzhou Second Hospital, Affiliated Hospital of Hangzhou Normal University: Prof Qiu Wusi, Miss Chen Mingmin. Tianjin Third Central Hospital, Department of Critical Care Medicine: Dr Lei Xu, Dr Chengfen Yin. Shanghai General Hospital, Shanghai Jiao Tong University School of Medicine, Department Of Critical Care Medicine: Dr Ruilan Wang, Dr Jinfeng Wang. The Second Hospital of Jilin University, Department Of Critical Care: Dr Yongjie Yin, Dr Min Zhang. Taizhou People S Hospital, Intensive Care Unit: Dr Jilu Ye, Dr Chungfang Hu. The First Affiliated Hospital of Nanjing Medical University, Department Of Geriatrics Intensive Care Unit: Dr Suming Zhou, Dr Min Huang. Zhejiang Hospital, Intensive Care Unit: Prof Jing Yan, Dr Yan Wang. Henan Provincial People S Hospital, Department of Critical Care Medicine: Dr Bingyu Qin, Dr Ling Ye. Qingdao Municipal Hospital, Intensive Care Unit: Dr Xie Weifeng. The Second Hospital of Lanzhou University, Department Of Critical Care Medicine: Dr Li Peije, Dr Nan Geng.

**Hong Kong**

*National Coordinator:* Dr. Lowell Ling

*Participating ICUs:* The Chinese University of Hong Kong, Prince of Wales Hospital, Department Of Anaesthesia And Intensive Care: Dr Lowell Ling.

**Japan**

*National Coordinator:* Dr. Yoshiro Hayashi

*Participating ICUs:* Kameda Medical Center, Department of Intensive Care Medicine: Dr Yoshiro Hayashi, Dr Toshiyuki Karumai. University Hospital Kyoto Prefectural University of Medicine, Intensive Care Unit: Dr Masaki Yamasaki, Dr Satoru Hashimoto. Hiroshima University Hospital, ICU: Dr Koji Hosokawa. Yokosuka General Hospital Uwamachi, Critical Care Medicine: Dr Jun Makino. Tokyo Metropolitan Tama Medical Center, Emergency and Critical Care Center: Dr Takeo Matsuyoshi. Kurashiki Central Hospital, Emergency Intensive Care Unit: Dr Akira Kuriyama. Tokyo Medical and Dental University, Department of Intensive Care Medicine: Dr Hidenobu Shigemitsu, Dr Yuka Mishima, Dr Michio Nagashima. St. Marianna University School of Medicine Hospital, Mixed ICU: Dr Hideki Yoshida, Prof. Shigeki Fujitani. Osaka City General Hospital, Emergency and Critical Care Medical Hospital: Dr Koichiro Omori, Dr Hiroshi Rinka. St. Marianna University School of Medicine, Yokohama City Seibu Hospital, Mixed ICU: Dr Hiroki Saito, Dr Kaori Atobe. Yokohama City University Hospital, Infection Prevention and Control Department: Dr Hideaki Kato. Yokohama City University Hospital, Intensive Care Department: Dr Shunsuke Takaki.

**Malaysia**

*National Coordinator:* Dr. Helmi Sulaiman

*Participating ICUs:* University Malaya Medical Centre, Department of Anaesthesiology and Intensive Care: Dr M. Shahnaz Hasan, Dr Muhamad Fadhil Hadi Jamaluddin. Hospital Tengku Ampuan Rahimah, Anaesthesia and Intensive Care: Dr Lee See Pheng, Dr Sheshendrasurian Visvalingam. Hospital Sarikei, Anaesthesiology & Intensive Care Unit: Dr Mun Thing Liew, Dr Siong Ling Danny Wong. Queen Elizabeth 1 Hospital, Department of Anaesthesiology and Intensive Care: Dr Kean Khang Fong, Dr Hamizah Bt Abdul Rahman. Hospital Serdang, Cardiothoracic and Perfusion Unit: Dr Zuraini Md Noor, Dr Lee Kok Tong. Hospital Tuanku Fauziah, Intensive Care Unit: Dr Abd. Hamid Azman. School Of Medical Sciences Universiti Sains Malaysia, Department of Anaesthesiology and Intensive Care: Dr Mohd Zulfakar Mazlan. Hospital Universiti Sains Malaysia, Department of Anaesthesiology and Intensive Care: Dr Saedah Ali.

**Philippines**

*National Coordinator:* Dr. Aaron Mark Hernandez

*Participating ICUs:* The Medical City Ortigas, Intensive Care Unit: Dr Anton Abello.

**Republic Of Korea**

*National Coordinator:* Dr Kyeongman Jeon

*Participating ICUs:* Samsung Medical Center, Medical Icu: Dr Kyeongman Jeon. Seoul National University Hospital, Medical Icu: Dr Sang-Min Lee. Hallym University Sacred Heart Hospital, Micu: Dr Sunghoon Park. Micu, Chonbuk National University Hospital: Prof Dr Seung Yong Park. Seoul National University Bundang Hospital, Medical Icu: Dr Sung Yoon Lim.

**Singapore**

*National Coordinator:* A/Prof Andrea Lay Hoon Kwa, Dr Qing Yuan Goh

*Participating ICUs:* Singapore General Hospital, Surgical Intensive Care Unit: Dr Qing Yuan Goh, A/Prof Shin Yi Ng. Singapore General Hospital, Neurosurgical Intensive Care Unit: Dr Sui An Lie, A/Prof Andrea Lay Hoon Kwa. Singapore General Hospital, Medical Intensive Care Unit: Dr Ken Junyang Goh. National University Hospital System Medical Intensive Care Unit: Dr Andrew Yunkai Li. Tan Tock Seng Hospital, Surgical Intensive Care Unit, Neurological Intensive Care Unit: Adj Asst Prof Caroline Yu Ming Ong, Dr Jia Yan Lim. Changi General Hospital, Medical Intensive Care Unit: Dr Jessica Lishan Quah, Dr Kangqi Ng. Changi General Hospital, Surgical Intensive Care Unit: Dr Louis Xiang Long Ng.

**Taiwan**

*National Coordinator:* Dr. Tony Yu-Chang Yeh

*Participating ICUs:* National Taiwan University Hospital, Sicu: Dr Yu Chang Yeh, Dr Nai-Kuan Chou. National Cheng Kung University Hospital, Division of Critical Care Medicine, Department of Internal Medicine: Dr Cong-Tat Cia. Mackay Memorial Hospital, Department of Critical Care Medicine: Dr Ting-Yu Hu, Dr Li-Kuo Kuo. National Taiwan University Hospital, Department of Internal Medicine, Micu: Dr Shih-Chi Ku.

**Thailand**

*National Coordinator:* Prof (Associate) Phunsup Wongsurakiat

*Participating ICUs:* Siriraj Hospital, Mahidol University, Critical Respitarory Care Unit, Department of Medicine: Prof (Associate) Phunsup Wongsurakiat. Vajira Hospital, Department of Internal Medicine: Dr Yutthana Apichatbutr, Dr Supattra Chiewroongroj.

**Middle East and North Africa**

**Dubai**

*National Coordinator:* Dr. Adel Alsisi

*Participating ICUs:* Dubai Hospital, Icu Department: Dr Rashid Nadeem, Dr Ashraf El Houfi.

**Egypt**

*National Coordinator:* Dr. Adel Alsisi

*Participating ICUs:* Cairo University Hospital (Qasr Al Ainy), Critical Care Department: Dr Adel Alsisi, Dr Amr Elhadidy, Dr Mina Barsoum.Medical Research Institute, Alexandria University, Biomedical Informatics and Medical Statistics (Icu): Dr Nermin Osman. Tanta University Hospital, Anaesthesia and Critical Care Department: Dr Tarek Mostafa. Tanta University Faculty of Medicine, Emergency Medicine and Traumatology Department: Dr Mohamed Elbahnasawy. Tanta University Emergency Hospital, Emergency, And Traumatology Department Critical Care Unit: Dr Ahmed Saber. Nasr City Health Insurance Hospital, Medical Icu: Dr Amer Aldhalia. Wingat Royal Hospital, Wingat Icu: Dr Omar Elmandouh. Elsahel Teaching Hospital, Icu: Dr Ahmed Elsayed. Ain Shams University Hospitals, Department of General Surgery: Dr Merihan A. Elbadawy, Dr Ahmed K. Awad. Alexandria Faculty of Medicine, Dialysis Intensive Care Unit: Miss Hanan M. Hemead.

**Iran**

*National Coordinator:* Prof. Farid Zand

*Participating ICUs:* Shiraz University of Medical Sciences, Anesthesiology and Critical Care Research Center: Prof Farid Zand, Dr Maryam Ouhadian. Ahvaz Jundishapur University of Medical Sciences, Air Pollution and Respiratory Diseases Research Center: Dr Seyed Hamid Borsi,, Dr Zahra Mehraban. Ahvaz Jundishapur University of Medical Sciences, Neurology Department: Dr Davood Kashipazha. Ahvaz Jundishapur University of Medical Sciences, Infectious and Tropical Diseases Research Center, Health Research Institute: Dr Fatemeh Ahmadi. Ahvaz Jundishapur University of Medical, Pain Research Center: Dr Mohsen Savaie, Dr Farhad Soltani, Dr Mahboobeh Rashidi, Dr Reza Baghbanian, Dr Fatemeh Javaherforoosh, Dr Fereshteh Amiri. Ahvaz Jundishapur University of Medical Sciences, Neurosurgery Department, Dr Arash Kiani. Ahvaz Jundishapur University of Medical Sciences, General Surgery Department, Dr Mohammad Amin Zargar. Tabriz University of Medical Sciences, Research Center for Integrative Medicine in Aging, Aging Research Institute: Prof Ata Mahmoodpoor. Jahrom University of Medical Sciences, Peimanieh Hospital : Dr Fatemeh Aalinezhad. Shiraz University of Medical Sciences, Shahid Rajaee Trauma Hospital : Dr Gholamreza Dabiri. Shiraz University of Medical Sciences, Trauma Research Center, Shahid Rajaee Hospital : Dr Golnar Sabetian, Dr Hakimeh Sarshad. Shiraz University of Medical Sciences, Anesthesiology and Critical Care Research Center: Dr Mansoor Masjedi, Dr Ramin Tajvidi. Zahedan University of Medical Sciences, Anesthesiology and Critical Care Department: Dr Seyed Mohammad Nasirodin (S.M.N.) Tabatabaei.

**Iraq**

*Participating ICUs:* Ibn Zuhur Hospital, Icu: Dr Abdullah Khudhur Ahmed.

**Israel**

*National Coordinator:* Prof. Pierre Singer

*Participating ICUs:* Rabin Medical Center Beilinson Hospital, General Intensive Care: Prof Pierre Singer, Dr Ilya Kagan, Dr Merav Rigler. Shaare Zedek Medical Center, Intensive Care Unit: Dr Daniel Belman, Dr Phillip Levin.

**Jordan**

*Participating ICUs:* Abdali Hospital, Icu: Dr Belal Harara, Dr Adei Diab.

**Lebanon**

*National Coordinator:* Dr Fayez Abillama

*Participating ICUs:* Lebanese American University Medical Center Rizk Hospital, Intensive Care: Dr Fayez Abilama, Dr Rebecca Ibrahim, Dr Aya Fares.

**Libya**

*National Coordinator:* Dr. Muhammed Elhadi

*Participating ICUs:* Aljalla Benghazi Center, Micu: Dr Ahmad Buimsaedah. Almokhtar Clinic, Intensive Care Unit: Dr Marwa Gamra. Althawra Central Hospital, Intensive Care Unit: Dr Ahmed Aqeelah. Brega General Hospital Bgh Libya, Icu: Dr Almajdoub Ali Mohammed Ali, Dr Ahmed Gaber Sadik Homaidan. National Heart Institute, Micu: Dr Bushray Almiqlash, Dr Hala Bilkhayr. Tobruk Medical Centre, Medical Icu: Dr Ahmad Bouhuwaish, Dr Ahmed Sa Taher. Tripoli Central Hospital, Icu: Dr Eman Abdulwahed, Dr Fathi A Abousnina, Dr Aisha Khaled Hdada. Tripoli Central Hospital, Unit C: Dr Rania Jobran. Zliten Medical Center, Icu of Zliten Medical Center: Dr Hayat Ben Hasan, Dr Rabab Shaban Ben Hasan.

**Morocco**

*National Coordinator:* Prof. Khalid Abidi

*Participating ICUs:* Avicenne Military Hospital, Icu: Dr Issam Serghini, Pr Rachid Seddiki. CHU Hassan II Fès, Intensive Care Unit A4: Dr Brahim Boukatta, Dr Nabil Kanjaa. Hospital Of Specialties, Critical Care Unit of Neurology and Neurosurgery: Prof Doumiri Mouhssine, Prof Maazouzi Ahmed Wajdi. Ibn Sina University Hospital, faculty of Medicine and Pharmacy, Mohammed V University in Rabat, Medical Icu: Pr Tarek Dendane, Pr Amine Ali Zeggwagh. Mohammed VI University Hospital of Oujda, Faculty Of Medicine and Pharmacy Oujda, Mohammed Premier University, Anesthesia and Resuscitation Department: Prof Brahim Housni, Dr Oujidi Younes. Mohammed VI University Hospital, Medical Icu, Marrakech: Prof Abdelhamid Hachimi. National Institute of Oncology of Rabat, Intensive Care Unit: Prof A Ghannam, Prof Z Belkhadir.

**Palestine**

*Participating ICUs:* ICU, Alia governmental hospital, Hebron / West Bank, Palestine: Dr. Sarah Amro. Gaza city, Alshifaa hospital, Gaza, Palestine: DR. Mustafa Abu Jayyab.

**Qatar**

*National Coordinator:* Dr Ali Aithssain

*Participating ICUs:* Hamad General Hospital, Medical Icu: Dr Ali Ait Hssain, Dr Abdurahaman Elbuzidi. Al Wakrah Hospital, Critical Care: Dr Edin Karic. Hamad General Hospital, Sicu: Dr Marcus Lance, Dr Shaikh Nissar.

**Saudi Arabia**

*Participating ICUs:* King Faisal Specialist Hospital &Research Center, Adult Critical Care Medicine: Dr Hend Sallam. Prince Sultan Medical Military Center, Intensive Care Unit: Dr Omar Elrabi, Dr Ghaleb A Almekhlafi. Security Force Hospital - Riyadh, Critical Care Unit: Dr Maher Awad, Dr Ahmed Aljabbary.

**Syria**

*Participating ICUs:* Al Mouwasat University Hospital, Icu: Dr Mohammad Karam Chaaban. Assad University Hospital, Neurological Intensive Care Unit: Dr Natalia Abu-Sayf. Damascus University Cardiac Surgery Hospital Near Al-Mouwasat University Hospital, Mazzeh Kiwan, Cardiac Surgery Icu: Dr Mohammad Al-Jadaan, Miss Lubna Bakr.

**Tunisia**

*National Coordinator:* Dr Mounir Bouaziz

*Participating ICUs:* Habib Bourguiba University Hospital, Department of Intensive Care: Dr Mounir Bouaziz, Dr Olfa Turki. Military Hospital of Tunis, Department of Anesthesiology And Intensive Care Unit, Lr12dn01: Pr Walid Sellami.

**Latin America and The Caribbean**

**Argentina**

*National Coordinator:* Dr. Gabriela Vidal

*Participating ICUs:* Hcas Cuenca Alta, Terapia Intensiva: Dr Pablo Centeno, Lic Natalia Morvillo. Hospital Central De Formosa, Servicio De Terapia Intensiva: Dr José Oscar Acevedo, Dr Patricia Mabel Lopez. Hospital Español De Mendoza, Terapia Intensiva De Adultos: Dr Rubén Fernández, Dr Matías Segura. Hospital Zatti, Ucia: Dra Marta Aparicio, Microbiologa Irene Alonzo. Instituto De Diagnostico De La Plata, Unidad De Terapia Intensiva: Dr Yanina Nuccetelli, Dr Pablo Montefiore.

**Colombia**

*National Coordinator:* Mario Arias

*Participating ICUs:* Clinica Universidad De La Sabana, Critical Care Unit : Dr Luis Felipe Reyes. Universidad De La Sabana, Infectious Diseases Department: Dr Luis Felipe Reyes.

**Mexico**

*National Coordinator:* Dr Silvio A. Ñamendys-Silva

*Participating ICUs:* Hospital Medica Sur, Department of Critical Care Medicine: Dr Silvio A. Ñamendys-Silva, Dr Juan P. Romero-Gonzalez. Centenario Hospital Miguel Hidalgo, Centenario Hospital Miguel Hidalgo: Dr Mariana Hermosillo, Dr Roberto Alejandro Castillo. Hospital General De Zona 14, Intensive Care Unit: Dr Jesús Nicolás Pantoja Leal, Dr Candy Garcia Aguilar. Hospital General Regional No.1, IMSS Tlaxcala: Dr Mara Ocotlan Gonzalez Herrera, Dr Missael Vladimir Espinoza Villafuerte. Hospital H+ Queretaro, Unidad De Terapia Intensiva Adultos: Dr Manuel Lomeli-Teran. Instituto Nacional de Ciencias Medicas y Nutricion Salvador Zubiran, Division of Pulmonary, Anesthesia and Critical Care Medicine: Dr Jose G. Dominguez-Cherit, Dr Adrian Davalos-Alvarez, Dr Silvio A. Ñamendys-Silva. UMAE Hospital de Especialidades Antonio Fraga Mouret, Centro Médico Nacional La RazaIMSS, Terapia Intensiva Hospital de Especialidades CMN La Raza: Dr Luis Sánchez-Hurtado, Dr Brigitte Tejeda-Huezo. Hospital General San Juan del Rio, Querétaro, , Unidad de Terapia Intensiva de Adultos: Dr Orlando R Perez-Nieto, Dr Ernesto Deloya Tomas.

**Europe And Central Asia**

**Belgium**

*National Coordinator:* Dr. Liesbet De Bus

*Scientific Committee:* Prof. Jan De Waele

*Recruitment of participating ICUs worldwide*: Mr. Guy Francois

*Participating ICUs:* Ghent University Hospital, Intensive Care Unit: Dr Liesbet De Bus, Dr Jan De Waele. A.S.Z., Iz: Dr Isabelle Hollevoet. Az Nikolaas, Icu: Dr Wouter Denys. Az Sint-Jan Av Brugge - Oostende Campus Brugge, Icu: Dr Marc Bourgeois. Az Sint-Lucas, Department of Intensive Care: Dr Sofie F.M. Vanderhaeghen. Centre Hospitalier De Jolimont, Soins Intensifs : Dr Jean-Baptiste Mesland, Dr Pierre Henin. Chu Ambroise Paré, Unité Des Soins Intensifs : Dr Lionel Haentjens. Chu Charleroi, Medico-Surgical Icu: Dr Patrick Biston, Mrs Cindérella Noel. Chu Liège, Soins Intensifs : Dr Nathalie Layos, Dr Benoît Misset. Clinique Saint-Pierre, Intensive Care Unit : Dr Nicolas De Schryver, Dr Nicolas Serck. Cliniques Universitaires Saint-Luc, UCLouvain, Soins Intensifs : Dr Xavier Wittebole. Uzbrussel, Intensieve Zorgen: Prof Elisabeth De Waele, Mrs Godelive Opdenacker.

**Bosnia And Herzegovina**

*National Coordinator:* Dr Pedja Kovacevic

*Participating ICUs:* University Clinical Centre of The Republic Of Srpska, Medical Intensive Care Unit: Dr Pedja Kovacevic, Dr Biljana Zlojutro.

**Croatia**

*National Coordinator:* Dr Ina Filipovic-Grcic

*Participating ICUs:* General Hospital Dubrovnik, Anesthesiology, And Intensive Care: Dr Aida Custovic, Dr Ina Filipovic-Grcic. University Hospital Centre Zagreb, Medical Intensive Care Unit: Prof Radovan Radonic, Dr Ana Vujaklija Brajkovic. University

Hospital Dubrava, Clinical Department of Anesthesiology, Reanimatology and Intensive Care: Prof Jasminka Persec, Dr Sanja Sakan, Dr Mario Nikolic, Dr Hrvoje Lasic.

**France**

*National Coordinator:* Prof. Marc Leone

*Scientific Committee:* Prof. Jean-François Timsit, Prof. Etienne Ruppe, Mr. Stephane Ruckly, Prof. Philippe Montravers

*Participating ICUs:* Hôpital Nord, Réanimation Polyvalente et Traumatologique : Pr Marc Leone, Dr Charlotte Arbelot. Bichat Claude Bernard, Réanimation Médicale et Infectieuse : Prof Jean-François Timsit, Mme Juliette Patrier. Bichat-Claude Bernard Hospital, Ap-Hp, Anesthesiology And Critical Care Medicine Department, Dmu Parabol: Dr N.Zappela, Pr P. Montravers. Centre Hospitalier De Bigorre, Service De Réanimation Polyvalente : Dr Thierry Dulac, Dr Jérémy Castanera. Centre Hospitalier De Cholet, Réanimation Polyvalente : Dr Johann Auchabie, Dr Anthony Le Meur. Centre Hospitalier De Dieppe, Médecine Intensive Réanimation : Dr A. Marchalot, Dr M. Beuzelin. Centre Hospitalier De Pau, Réanimation Polyvalente : Dr Alexandre Massri, Dr Charlotte Guesdon. Ch Annecy Genevois, Réanimation Polyvalente : Dr Etienne Escudier. Ch De Charleville-Mézières, Médecine Intensive Réanimation : Dr Philippe Mateu, Dr Jérémy Rosman. Ch Tourcoing, Service De Reanimation: Dr Olivier Leroy, Dr Serge Alfandari. Chu Compiegne Noyon, Réanimation : Dr Alexandru Nica. Chu Gabriel Montpied, Médecine Intensive Et Réanimation : Dr Bertrand Souweine, Dr Elisabeth Coupez. Chu Lille, Hôpital Roger Salengro, Pôle De Réanimation : Dr Thibault Duburcq. Chu Lille, Surgical Critical Care, Department of Anesthesiology and Critical Care: Prof Eric Kipnis, Dr Perrine Bortolotti. Chu Rennes, Service De Maladies Infectieuses Et Réanimation Médicale : Dr Mathieu Le Souhaitier. Cochin, Medecine Intensive Reanimation: Dr Jean-Paul Mira. Ghef Site De Marne-La-Vallée, Réanimation Polyvalente : Dr Pierre Garcon, Dr Matthieu Duprey. Groupe Hospitalier Nord Essonne - Site Longjumeau, Réanimation Polyvalente : Dr Martial Thyrault, Dr Rémi Paulet. Groupe Hospitalier Paris Saint Joseph, Médecine Intensive et Réanimation : Dr François Philippart, Dr Marc Tran, Dr Cédric Bruel. Hôpital Beaujon, Department of Anesthesiology and Critical Care: Dr Emmanuel Weiss, Dr Sylvie Janny, Dr Arnaud Foucrier. Hopital De Gui De Chauliac, Departement Anesthesie Reanimation Gui De Chauliac : Dr Pierre-François Perrigault, Dr Flora Djanikian. Hôpital De La Source, Centre Hospitalier Régional D'orléans, Médecine Intensive & Réanimation (Medical Icu): Dr François Barbier. Hôpital De La Timone, Médecine Intensive Réanimation : Dr Marc Gainnier, Dr Jérémy Bourenne. Hopital De Mercy, Chr Metz-Thionville, Service De Réanimation Polyvalente Et Usc: Dr Guillaume Louis. Hopital Du Scorff, Service De Réanimation : Dr Roland Smonig. Hôpital Edouard Herriot, Médecine Intensive-Réanimation : Dr Laurent Argaud, Dr Thomas Baudry. Hôpital Henri Mondor, Service De Réanimation Médicale : Pr Armand Mekonted Dessap, Dr Keyvan Razazi. Hôpital Louis Pasteur, Réanimation : Dr Pierre Kalfon, Mr Gaëtan Badre. Montpellier University Hospital, Intensive Care Medicine Lapeyronie Hospital: Dr Romaric Larcher. Nimes University Hospital, Service Des Réanimations : Prof Jean-Yves Lefrant, Dr Claire Roger. Purpan, Réanimation Polyvalente : Dr Benjamine Sarton, Dr Stein Silva. Sorbonne Universite Pitie Salpetriere, Médecine Intensive Et Réanimation Neurologique : Dr Sophie Demeret, Dr Loïc Le Guennec. Sud Essonne Hospital, Department of Intensive Care Medicine: Dr Shidasp Siami, Mrs Christelle Aparicio. Tenon Hospital, Service De Médecine Intensive Réanimation : Dr Guillaume Voiriot, Dr Muriel Fartoukh. University Hospital Of Poitiers, Surgical And Neuro Intensive Care Units: Dr Claire Dahyot-Fizelier, Dr Nadia Imzi. University Of Montpellier, Phymedexp Inserm Cnrs: Dr Kada Klouche.

**Germany**

*National Coordinator:* Prof. Hendrik Bracht

*Participating ICUs:* University Hospital Ulm, Icu G1: Dr Hendrik Bracht, Dr Sandra Hoheisen. Jena University Hospital, Dept. Of Anesthesiology and Intensive Care Medicine: Dr Frank Bloos, Dr Daniel Thomas-Rueddel. Universitätsklinikum Leipzig, Medical Icu: Dr Sirak Petros, Dr Bastian Pasieka. University Hospital Heidelberg, Station 13 Iopsis: Dr Simon Dubler, Dr Karsten Schmidt. University Hospital Muenster, Department of Anesthesiology, Intensive Care Medicine and Pain Therapy: Dr Antje Gottschalk, Dr Carola Wempe. University Hospital of Saarland, Dept. Of Internal Medicine V - Pneumology, Allergology and Critical Care Medicine: Prof Philippe Lepper, Dr Carlos Metz.

**Kazakhstan**

*National Coordinator:* Dr. Dmitriy Viderman

*Participating ICUs:* University Medical Center, National Research Oncology Center, Intensive Care Unit: Dr Dmitriy Viderman, Dr Yerlan Umbetzhanov . Karaganda Medical University, Department of Emergency Medicine, Anesthesiology and Resuscitation Non-Commercial Joint-Stock Company: Associate Prof., Dr Miras Mugazov, Dr Yelena Bazhykayeva. Medical Center Hospital of The President's Affairs Administration of The Republic of Kazakhstan, Intensive Care Unit: Dr Zhannur Kaligozhin, Dr Baurzhan Babashev. National Research Oncology Center, Department of Oncohematological Resuscitation, Resuscitation, Intensive Care: Dr Yevgeniy Merenkov, Dr Talgat Temirov.

**Greece**

*National Coordinator:* Dr. Kostoula Arvaniti

*Participating ICUs:* Papageorgiou Hospital, Intensive Care Unit: Dr Kostoula Arvaniti, Dr Dimitrios Smyrniotis. Agioi Anargiroi Hospital, Agioi Anargiroi Icu: Dr Vasiliki Psallida, Dr Georgios Fildisis. G Papanikolaou General Hospital, 1st Icu: Dr Vasiliki Soulountsi, Dr Evangelos Kaimakamis. G Papanikolaou General Hospital, B Icu: Dr Cristina Iasonidou, Dr Sofia Papoti. General Hospital G. Gennhmatas, Gnth "G Gennhmatas": Dr Foteini Renta, Dr Maria Vasileiou. General Hospital of Athens G. Gennimatas, Icu: Dr Vasiliki Romanou, Dr Vasiliki Koutsoukou. Gh Imathia Veria, Icu: Dr Mariana Kristina Matei, Dr Leora Moldovan. Icu, Hygeia General Hospital: Dr Ilias Karaiskos, Dr Harry Paskalis. Intensive Care Unit, General Hospital of Giannitsa: Dr Kyriaki Marmanidou. Intensive Care Unit, Hippocration General Hospital Of Athens: Dr M. Papanikolaou, Dr C.Kampolis. Katerini General Hospital, Gnk Icu: Dr Marina Oikonomou, Dr Evangelos Kogkopoulos. Konstantopoulion-Patision Hospital, Icu: Dr Charikleia Nikolaou, Dr Anastasios Sakkalis. Mediterraneo Hospital, Icu/Hdu : Dr Marinos Chatzis, Dr Maria Georgopoulou. Saint Savvas Hospital, Icu: Dr Anna Efthymiou, Dr Vasiliki Chantziara. Sismanogleio Hospital, Sismanogleion Icu: Dr Aikaterini Sakagianni, Dr Zoi (Zoe) Athanasa (Athanassa). Theageneio Anticancer Hospital, Icu: Dr Eirini Papageorgiou, Dr Fadi Ali. University Hospital Attikon, National And Kapodistrian University Of Athens, Department Of Critical Care: Pr Georges Dimopoulos, Dr Mariota Panagiota Almiroudi. University Hospital Heraklion, Department of Intensive Care: Dr Polychronis Malliotakis, Dr Diamantina Marouli. University Hospital of Alexandroupolis, Department Of Intensive Care: Dr Vasiliki Theodorou, Dr Ioannis Retselas. University Hospital of Ioannina, Intensive Care Unit: Pr Vasilios Kouroulas, A/Pr Georgios Papathanakos.

**Italy**

*National Coordinator:* Prof. Matteo Bassetti and Dr. Daniele Giacobbe

*Participating ICUs:* Città Della Salute E Della Scienza - Molinette, Anestesia E Rianimazione Universitaria: Dr Giorgia Montrucchio, Dr Gabriele Sales. Fondazione Policlinico Universitario A. Gemelli Irccs. Universita Cattolica Del Sacro Cuore. Italy, Uoc Di Anestesia, Rianimazione, Terpia Intensiva E Tossicologia Clinica: Dr Gennaro De Pascale, Dr Luca Maria Montini, Dr Simone Carelli, Dr Joel Vargas, Ms Valentina Di Gravio. Irccs Ospedale Policlinico San Martino, U.O. Anestesia E Rianimazione: Prof Daniele Roberto Giacobbe, Dr Angelo Gratarola, Dr Elisa Porcile, Dr Michele Mirabella. Irccs Sacro Cuore Don Calabria, Terapia Intensiva: Dr Ivan Daroui, Dr Giovanni Lodi. Madonna Delle Grazie, U.O.C. Anestesia E Rianimazione: Dr Francesco Zuccaro, Dr Maria Grazia Schlevenin. Ospedale Policlinico San Martino, Irccs Per L’oncologia E Le Neuroscienze, Uo Clinica Anestesiologica E Terapia Intensiva: Prof Paolo Pelosi, Dr Denise Battaglini. Policlino Paolo Giaccone, Università Degli Studi Di Palermo, Terapia Intensiva Polivalente: Dr Andrea Cortegiani, Dr Mariachiara Ippolito, Dr Davide Bellina, Dr Andrea Di Guardo. Regina Elena National Cancer Institute of Rome, Anesthesia and Intensive Care Department: Dr Lorella Pelagalli, Dr Marco Covotta. Sant'andrea Hospital Sapienza University of Rome, Department of Medical And Surgical Science And Translational Medicine Intensive Care Unit: Dr Monica Rocco, Dr Silvia Fiorelli. University Hospital O.O.R.R., Department of Anesthesia And Intensive Care: Prof Antonella Cotoia, Dr Anna Chiara Rizzo.

**Poland**

*National Coordinator:* Dr Adam Mikstacki

*Participating ICUs:* Hospital In Puszczykowo, Poznan University of Medical Sciences, Department of Anaesthesiology and Intensive Therapy: Dr Adam Mikstacki, Dr Barbara Tamowicz. 10 Wojskowy Szpital Kliniczny, Oddzial Kliniczny Anestezjologii I Intensywnej Terapii: Dr Irmina Kaptur Komorowska, Dr Anna Szczesniak. Szpital Wojewodzki W Opolu, Oddzial Anestezjologii I Intensywnej Terapii: Dr Jozef Bojko, Dr Anna Kotkowska. Uck Wum, Oddzial Intensywnej Terapii (Icu ): Dr Paulina Walczak-Wieteska, Dr Dominika Wasowska. Wojewodzki Szpital Zespolony, Oddzial Anestezjologii I Intensywnej Terapii: Dr Tomasz Nowakowski, Dr Hanna Broda. Wss Im. Wl. Bieganskiego, Oddzial Anestezjologii I Intensywnej Terapii - Osrodek Pozaustrojowych Technik Wspomagania Czynnosci Nerek I Wątroby: Prof Assoc Mariusz Peichota, Dr Iwona Pietraszek-Grzywaczewska.

**Republic Of Ireland**

*National Coordinator:* Prof Ignacio Martin-Loeches

*Participating ICUs:* St Jame's Hospital, Intensive Care Unit: Prof Ignacio Martin-Loeches, Dr Alessandra Bisanti.

**Portugal**

*National Coordinator: Prof. José Artur Paiva*

*Scientific Committee: Prof. Pedro Póvoa*

*Participating Icus: Centro Hospitalar Medio Tejo - Unidade Abrantes, Ucip: Dr Nuno Cartoze, Dr Tiago Pereira. Centro Hospitalar Universitário do Porto, Sci 1: Dr Nádia Guimarães, Dr Madalena Alves. Centro Hospitalar Vila Nova De Gaia/Espinho, Unidade De Cuidados Intensivos Polivalente: Dr Ana Josefina Pinheiro Marques, Dr Ana Rios Pinto. CHUA Faro, Smi-1 : Dr Andriy Krystopchuk, Dr Ana Teresa. Hospital De Cascais Dr Jose De Almeida, Unidade de Cuidados Intensivos: Dr António Manuel Pereira de Figueiredo, Dr Isabel Botelho. Hospital Curry Cabral, Intensive Care Medicine Department: Dr Tiago Duarte. Hospital Sao Francisco Xavier, CHLO, Unidade De Cuidados Intensivos Polivalente: Dr Vasco Costa, Dr Rui Pedro Cunha. Hospital Pedro Hispano, Serviço De Medicina Intensiva: Dr Elena Molinos, Dr Tito da Costa. CHULC, Hospital Sao José, Unidade de Urgência Médica: Dr Sara Ledo, Dr Joana Queiró. ULS Litoral Alentejano, Serviço de Medicina Intensiva: Dr Dulce Pascoalinho. ULS Nordeste, Unidade de Cuidados Intensivost: Dr Cristina Nunes. ULSAM, UCI: Dr José Pedro Moura, Dr Énio Pereira. ULS Baixo Alentejo, Unidade Cuidados Intensivos Polivalente: Dr António Carvalho Mendes.*

**Romania**

*National Coordinator:* Dr Liana Valeanu

*Participating ICUs:* Emergency Institute for Cardiovascular Diseases Prof. Dr. C. C. Iliescu, 1st Anesthesia and Intensive Care Department: Dr Liana Valeanu, Prof Serban Bubenek-Turconi. Clinical Emergency Hospital Bucharest, Anesthesia and Intensive Care Department: Prof Ioana Marina Grintescu, Dr Cristian Cobilinschi. Emergency Institute for Cardiovascular Diseases Prof. Dr. C. C. Iliescu, 2nd Anesthesia and Intensive Care Department: Prof Daniela Carmen Filipescu, Dr Cornelia Elena Predoi. Fundeni Clinical Institute, 3rd Department of Anesthesia and Intensive Care: Prof Dana Tomescu, Dr Mihai Popescu, Dr Alexandra Marcu. University Of Medicine and Pharmacy “Grigore T Popa”, Anesthesia and Intensive Care Department: Prof Ioana Grigoras, Dr Olguta Lungu.

**Russian Federation**

*National Coordinator:* Prof. Alexey Gritsan

*Participating ICUs:* V.F. Voino-Yasenetsky Krasnoyarsk State Medical University, Krasnoyarsk Regional Clinical Hospital, Dep. Anaesthesiology and Intensive Care #3: Prof Alexey Gritsan. City Clinical N.I.Pirogov Hospital, Clinical Pharmacology: Dr Anastasia Anderzhanova, Dr Yulia Meleshkina. City Clinical N.I.Pirogov Hospital, Icu: Dr Marat Magomedov. E.A. Vagner Perm State Medical University, Intensive Care Unit: Prof Nadezhda Zubareva, Dr Maksim Tribulev. Krasnoyarsk Regional Clinical Hospital, Dep. Anaesthesiology and Intensive Care #3: Dr Denis Gaigolnik. Petrovsky National Research Centre of Surgery, Intensive Care: Dr Aleksandr Eremenko, Dr Natala Vistovskaya, Dr Maria Chukina. Privolzhskiy District Medical Center, Department Anesthesiology and Intensive Care: Dr Vladislav Belskiy, Dr Mikhail Furman.

**Spain**

*National Coordinator:* Dr. Ricard Ferrer Rocca

*Participating ICUs:* Vall D'herbon, Intensive Care Medicine: Dr Ricard Ferrer Rocca, Dr Maria Martinez, Dr Vanessa Casares. Hospital Clinic De Barcelona, Surgical Icu: Dr Ricard Mellado Artigas. Hospital De La Santa Creu I Sant Pau, Intensive Care Unit : Dr Paula Vera, Dr Matias Flores. Hospital De Terrassa, Medicina Intensiva: Dr Joaquin Amador Amerigo. Hospital Del Mar, Critical Care Unit: Dr Maria Pilar Gracia Arnillas, Dr Rosana Munoz Bermudez. Hospital Germans Trias I Pujol, Critical Care Unit: Prof, Dr Fernando Armestar, Dr Beatriz Catalan, Dr Regina Roig, Dr Laura Raguer, Dr María Dolores Quesada. Hospital Parc Tauli, Icu: Dr Emilio Diaz Santos, Dr Gemma Gomà. Hospital Punta De Europa, Intensive Care Unit : Dr Alejandro Ubeda, Dra Maria Salgado. Hospital Universitario Central De Asturia, Uci-Huca: Dr Lorena Forcelledo Espina, Dr Emilio Garcia Prieto. Hospital Universitario La Paz, Intensive Care Unit, Servicio De Medicina Intensiva: Dra Mj Asensio, Dra M. Rodriguez. Hospital Universitario La Paz, Surgical Critical Care Unit : Dr Emilio Maseda, Dr Alejandro Suarez De La Rica. Hospital Universitarion Son Espases, Unidad De Cuidados Intensivos: Dr J Ignacio Ayestaran, Dr Mariana Novo. University Hospital Severo Ochoa, Intensive Care Unit: Dr Miguel Angel Blasco-Navalpotro, Dr Alberto Orejas Gallego.

**Sweden**

*National Coordinator:* Dr Fredrik Sjovall

*Participating ICUs:* Skane University Hospital, Intensive- And Perioperative Care: Dr Fredrik Sjövall, Dr Dzana Spahic. Ostra Sjukhuset Sahlgrenska University Hospital, Anopiva: Dr Carl Johan Svensson. Umeå University, Anesthesiology and Intensive Care Medicine, Surgical and Perioperative Sciences: Dr Michael Haney, Dr Alicia Edin. Universitetssjukhuset I Linkoping, Anopiva: Dr Joyce Åkerlund, Dr Lina De Geer.

**Switzerland**

*National Coordinator:* Dr. Josef Prazak

*Scientific Committee:* Dr. Niccolò Buetti

*Participating ICUs:* Inselspital, Bern University Hospital, Department of Intensive Care Medicine: Dr Josef Prazak, Dr Stephan Jakob. Chuv, Service De Médecine Intensive Adulte : Dr Jl Pagani, Mrs S Abed-Maillard.

**Turkey**

*National Coordinator:* Prof. Murat Akova, Dr. Abdullah Tarık Aslan

*Participating ICUs:* Hacettepe University of Faculty of Medicine, Intensive Care Unit(ICU): Dr Murat Akova, Dr Abdullah Tarik Aslan, Abdurrahman Yurtaslan Ankara Oncology Training and Research Hospital, Department of Anesthesiology: Dr Arif Timuroglu. Acibadem Fulya Hospital, Infectious Diseases: Dr Sesin Kocagoz, Dr Hulya Kusoglu. Acibadem Kadikoy Hospital, ICU: Dr Selcuk Mehtap, Dr Solakoğlu Ceyhun. Ankara University Faculty Medicine Ibni Sina Hospital, Medical ICU: Prof. Dr. Neriman Defne Altintas, Dr Leyla Talan. Ankara Yildirim Beyazit University, Ankara City Hospital, Infectious Diseases and Clinical Microbiology: Dr Bircan Kayaaslan, Dr Ayşe Kaya Kalem. Aydin Adnan Menderes University Research Hospital, Anesthesia and Reanimation ICU: Prof. Dr. Ibrahim Kurt, Dr (Professor) Murat Telli, Dr (Associate Professor) Barcin Ozturk. Baskent University Hospital, Infectious Diseases and Clinical Microbiology: Dr Çiğdem Erol. Bitlis Goverment Central Hospital, Bitlis Icu: Dr Emine Kubra Dindar Demiray, Dr Sait Çolak. Duzce University Hospital, Medical ICU: Dr Türkay Akbas. Erciyes University, ICU: Prof. Dr. Kursat Gundogan, Dr Ali Sari. Fatih Sultan Mehmet Research and Training Hospital, Infection Diseases: Dr Canan Agalar, Dr Onur Çolak. Hitit University Erol Olcok Education and Research Hospital, Infectious Diseases and Clinical Microbiology: Prof. Dr. Nurcan (N) Baykam, Assistant Prof. Dr Ozlem (O) Akdogan. Istanbul Medipol University, Kosuyolu Hospital, Infectious Diseases and Clinical Microbiology: Dr Mesut Yilmaz, Dr Burcu Tunay, Dr Rumeysa Cakmak. Istanbul University-Cerrahpasa, Cerrahpasa Medical Faculty, Sadi Sun ICU: Prof.Dr. Nese Saltoglu, Ass Prof.Dr. Ridvan Karaali. Karadeniz Technical University Faculty of Medicine, Infectious Disease and Clinical Microbiology: Prof Dr. Iftihar Koksal, Assist. Prof. Firdevs Aksoy. Karadeniz Technical University Farabi Hospital, Anesthesia ICU 1: Dr Ahmet Eroglu. Kartal Dr. Lutfi Kirdar Training and Research Hospital, ICU: Dr Kemal Tolga Saracoglu, Dr Yeliz Bilir. Kayseri City Hospital, ICU: Dr Seda Guzeldag. Mersin University Hospital, Department of Infectious Diseases and Clinical Microbiology: Dr Gulden Ersoz, Dr Guliz Evik. Pamukkale Univ, Anesthesiology and Reanimation: Prof Hulya Sungurtekin, Dr Cansu Ozgen. School Of Medicine, Medipol Mega University Hospitals Complex, Department of Anesthesiology and Reanimation: Dr Cem Erdoğan. University of Health Sciences Diskapi Yildirim Beyazit Training and Research Hospital, The Department of Infectious Diseases and Clinical Microbiology and ICU: Dr Yunus Gürbüz, Dr Nilgün Altin. Turgut Ozal Medical Center, Department of Infectious Diseases and Clinical Microbiology: Dr Yasar Bayindir, Dr Yasemin Ersoy. University of Health Sciences Istanbul Umraniye Training and Research Hospital, Anaestesia and Reanimation: Dr Senay Goksu, Dr Ahmet Akyol. University of Health Sciences, Kartal Dr. Lutfi Kirdar Training and Research Hospital, Infectious Diseases and Clinical Microbiology: Prof Ayse Batirel, Dr Sabahat Cagan Aktas.

**The United Kingdom**

*National Coordinator:* Dr. Andrew Conway Morris

*Participating ICUs:* Addenbrookes Hospital, John V Farman Intensive Care Unit: Dr Andrew Conway Morris, Dr Matthew Routledge. Addenbrookes Hospital, Neurocritical Care Unit (NCCU): Dr Andrew Conway Morris, Dr Ari Ercole. Charing Cross Hospital - Imperial College NHS Trust, Intensive Care Unit, Level 11: Dr David Antcliffe, Ms Roceld Rojo. Countess Of Chester Foundation Trust, Intensive Care Unit: Dr Kate Tizard, Dr Maria Faulkner. Darlington Memorial Hospital Intensive Care Unit, County Durham and Darlington NHS Foundation Trust: Dr Amanda Cowton, Dr Melanie Kent. Croydon University Hospital, Critical Care Unit: Dr Ashok Raj, Dr Artemis Zormpa, Dr George Tinaslanidis, Mrs Reena Khade. Department Of Anaesthetics and Intensive Care Medicine, Queen Elizabeth Hospital Birmingham: Dr Tomasz Torlinski, Dr Randeep Mulhi, Dr Shraddha Goyal, Dr Manan Bajaj, Dr Marina Soltan, Dr Aimee Yonan, Dr Rachael Dolan. Department Of Microbiology, Queen Elizabeth Hospital Birmingham: Dr Aimee Johnson. Freeman Hospital, ICCU 37: Dr Caroline Macfie, Dr James Lennard. Hammersmith Hospital - Imperial College NHS Trust, Intensive Care Unit, Level 11: Ms Maie Templeton, Ms Sonia Sousa Arias. James Cook University Hospital, Icu2/3: Dr Uwe Franke, Mr Keith Hugill. Medway Maritime Hospital, Intensive Care Unit: Mrs Hollie Angell. Ninewells Hospital and Medical School NHS Tayside, Intensive Care Unit: Dr Benjamin J Parcell, Dr Katherine Cobb, Dr Stephen Cole. North Cumbria University Hospitals NHS Trust, North Cumbria University Hospitals NHS Trust: Dr Tim Smith, Dr Clive Graham. North Manchester General Hospital, Critical Care Ward: Dr Jaroslav Cerman, Dr Allison Keegan. Queen Elizabeth Hospital, Gateshead Health NHS Foundation Trust, Critical Care Department: Mrs Jenny Ritzema, Mrs Amanda Sanderson. Queen Elizabeth Hospital, Lewisham and Greenwich NHS Trust, Critical Care Unit: Dr Ashraf Roshdy. Royal Gwent Hospital, Critical Care Unit: Dr Tamas Szakmany, Dr Tom Baumer. Royal London Hospital, Adult Critical Care Unit: Dr Rebecca Longbottom, Dr Daniel Hall. Royal Marsden NHS Foundation Trust, Critical Care Unit: Dr Kate Tatham, Dr S Loftus, Dr A Husain, Dr E Black, Dr S Jhanji, Dr R Rao Baikady. Royal Victoria Hospital, Belfast, Regional Intensive Care Unit: Dr Peter Mcguigan, Dr Rachel Mckee. Sandwell And West Birmingham Hospitals NHS Trust, Intensive Care Unit: Dr Santhana Kannan, Dr Supriya Antrolikar, Dr Nicholas Marsden. St Mary's Hospital - Imperial College NHS Trust, Intensive Care Unit, Level 11: Dr Valentina Della Torre, Ms Dorota Banach. Stepping Hill Hospital, Stepping Hill ICU: Dr Ahmed Zaki, Dr Matthew Jackson. University Hospitals of North Midlands, Critical Care Unit: Dr Moses Chikungwa. Warwick Hospital, Intensive Care Unit: Dr Ben Attwood, Dr Jamie Patel. West Suffolk NHS Foundation Trust, Critical Care: Dr Rebecca E Tilley, Miss Sally K Humphreys. Wirral University Teaching Hospital, Intensive Care Unit: Dr Paul Jean Renaud.

**Ukraine**

*Participating ICUs:* Kharkiv Clinical Infectious Diseases Hospital, Intensive Care: Prof Anton Sokhan, Dr Yaroslava Burma.

**North America**

**Canada**

*National Coordinator:* Prof. Wendy Sligl

*Participating ICUs:* University of Alberta Hospital, General Systems Intensive Care Unit (Gsicu): Dr Wendy Sligl, Nadia Baig, Lorena McCoshen. Royal Alexandra Hospital, General Systems Intensive Care Unit (Gsicu): Dr Demetrios J Kutsogiannis, Dr Wendy Sligl, Patricia Thompson, Tayne Hewer.

**South Asia**

**Bangladesh**

*National Coordinator:* Dr Raihan Rabbani

*Participating ICUs:* General Icu, Dhaka: Dr Raihan Rabbani, Dr Shihan Mahmud Redwanul Huq. Asgar Ali Hospital, Critical Care Medicine: Dr Rajib Hasan, Dr Mohammad Motiul Islam.

**India**

*National Coordinator:* Prof. Mohan Gurjar

*Participating ICUs:* Sanjay Gandhi Postgraduate Institute of Medical Sciences (SGPGIMS), Lucknow, Department of Critical Care Medicine: Dr Mohan Gurjar, Dr Arvind Baronia. All India Institute of Medical Sciences (AIIMS) Jodhpur, Department of Anaesthesiology & Critical Care Medicine: Dr Nikhil Kothari, Dr Ankur Sharma. All India Institute of Medical Sciences (AIIMS), Pulmonary Medicine ICU, Department of Pulmonary Medicine: Dr Saurabh Karmakar, Dr Priya Sharma. Breach Candy Hospital Trust, SICU: Dr Janardan Nimbolkar, Dr Pratit Samdani. Cauvery Heart And Multi-Speciality Hospital, MICU: Dr Vaidyanathan R, Dr Noor Ahmedi Rubina. CHL Hospitals, Dept of Critical Care Services: Dr Nikhilesh Jain, Dr Madhumati Pahuja. Indira Gandhi Institute of Medical Sciences, Trauma & Emergency: Dr Ritu Singh, Dr Saurav Shekhar. King George's Medical University, Department of Critical Care Medicine: Dr Syed Nabeel Muzaffar, Dr Ahmad Ozair, Dr Suhail Sarwar Siddiqui. Medica Superspecialty Hospital, Medica Institute of Critical Care: Dr Payel Bose, Dr Avijatri Datta. Sir H N Reliance Foundation Hospital, Critical Care Unit: Dr Darshana Rathod, Dr Mayur Patel. Sri Ramachandra Institute of Higher Education and Research, Department of Critical Care Medicine: Prof MK Renuka, Dr Sailaja K Baby. St Johns Medical College Hospital, Department of Critical Care Medicine, MICU: Dr Carol Dsilva, Dr Jagadish Chandran. Tata Medical Center, Critical Care Medicine: Dr Pralay Ghosh, Dr Sudipta Mukherjee. Yashoda Hospital, Somajiguda, Hyderabad: Dr Kaladhar Sheshala, Dr Krushna Chandra Misra.

**Sub-Saharan Africa**

**Nigeria**

*National Coordinator:* Dr. Oyebola O. Adekola

*Participating ICUs:* Ahmadu Bello University Teaching Hospital Shika Zaria Abuth Zari, Icu Abuth Zaria: Dr Saidu Yusuf Yakubu, Dr Euphemia Mgbosoro Ugwu. Lagos University Teaching Hospital, Department of Anaesthesia And Intensive Care: Dr John (O) Olatosi, Dr Ibironke Desalu. One Life Hospital, One Life Intensive Care Unit: Dr Gabriel Asiyanbi, Dr Motunrayo Oladimeji. University College Hospital, Anaesthesia: Dr Olusola Idowu, Dr Fowotade Adeola.

**South Africa**

*National Coordinator:* Prof. Mervyn Mer

*Participating ICUs:* Charlotte Maxeke Johannesburg Academic Hospital, Ward 576: Prof Mervyn Mer, Mrs Melanie Mc Cree.

**Sudan**

*National Coordinator:* Dr. Bashir El Sanousi

*Participating ICUs:*Al-Rajhi Hospital, Medicine: Dr Ali Adil Ali Karar. East Nile Hospital, Intensive Care Unit: Dr Elfayadh Saidahmed, Dr Hytham K.S. Hamid.
